# The Computational Analysis of Protein Structures: Sources, Methods, Systems and Results

**DOI:** 10.6028/jres.094.011

**Published:** 1989

**Authors:** Arthur M. Lesk, Anna Tramontano

**Affiliations:** European Molecular Biology Laboratory, Heidelberg, F.R.G.; MRC Laboratory of Molecular Biology, Cambridge, U.K.; European Molecular Biology Laboratory, Heidelberg, F.R.G; International Institute of Genetics and Biophysics, via Marconi, 10, 80215, Naples, Italy

**Keywords:** data banks, molecular biology, molecular graphics, protein structures, structure prediction

## Abstract

Computational molecular biology is a relatively new specialty that has arisen in response to the very large amount and quality of data currently being produced, including gene and protein sequences (“one-dimensional” information) and nucleic acid and protein structures (“three-dimensional” information). Many important biological investigations can be carried out only through effective computational access to the entire corpus of data. This has stimulated the development of data banks and information retrieval systems. For example, after determination of a new gene sequence, one would like to know whether it is possible to say anything about its structure and function. To try to answer this question one screens the sequence of the corresponding protein for a significant similarity to a protein of known structure. In this article we shall describe the kinds of inferences that are possible if such a relationship is found.

## Introduction

The state of information-retrieval systems in molecular biology is currently undergoing rapid change. This is partly a result of the great increase in the sheer amount of data available, and partly the result of advances in computing equipment that have made available very powerful systems capable of supporting high-capacity information storage, demanding calculations, and complex real-time graphics, and a better definition of the roles in the partnership between the program system and the scientist. But it is also the result of our beginning to understand somewhat better the kinds of questions we want to ask. For example, until recently the one-dimensional world of sequence calculations and the three-dimensional world of structure calculations remained aloof from each other; now it is recognized that it is essential to bring both sets of data to bear on problems together. For another example, until recently many people—in the three-dimensional world—would work on a single protein structure or family of structures in isolation. Now we recognize the importance of free and common access to all available proteins, because we can recognize structural themes common to a wide variety of structures.

These three factors—large and rapidly increasing amounts of data, new powerful computer systems, and greater sophistication—are now in collision. Here we shall describe what might emerge.

## The Data

Nucleotide sequences contain the blueprints for the development of living organisms. They directly encypher the amino acid sequences of proteins, agents of biological structure and function. Once the amino acid sequence of a protein has been synthesized, it then spontaneously folds to create a unique three-dimensional protein conformation. It is at this point that the linear genetic code is translated into three dimensions.

Nucleic acid sequences, protein sequences, and protein structures are all collected and distributed by data banks.

Nucleic acid sequences are collected by a tripartite association of organizations: GenBank® in the United States of America, with scientists at Los Alamos National Laboratory and Intelligenetics, Inc.; The Nucleotide Sequence Data Bank at the European Molecular Biology Laboratory in Heidelberg, Federal Republic of Germany; and the DNA Data Bank of Japan, at the National Institute of Genetics, in Mishima. These groups collaborate in harvesting data from published journals, and in sharing the results. To an increasing extent, the data banks are receiving data in computer-readable form directly from scientists. The data are converted to standard formats, checked and annotated, and then exchanged among the databanks and distributed to scientists.

It may be interesting to have some standards of comparison for the amounts of data involved. If one base pair is stored as one byte, the genome of the Epstein-Barr virus has 172 kbytes, the genome of the much studied bacterium *E. coli* has 4000 kbytes, the genome of yeast is around 20,000 kbytes, and the human genome is a factor of 1000 above *E. coli* at 4 × 10^9^ bases or 4 × 10^6^ kbytes. The *E. coli* genome stored at 1 byte per base pair has approximately the same number of characters as the Cambridge, England telephone directory. A human genome has about an order of magnitude more characters than the Oxford English Dictionary. The Dictionary in its new printed form is a set of 16 large volumes, and also occupies an entire compact disc.

Protein sequences are collected by another triple partnership. For many years, the group at the National Biomedical Research Foundation in Washington, D.C. maintained the major computerreadable archive of protein sequence data. In addition to collecting, annotating, and distributing sequences, this group has developed a powerful information retrieval system integrated with the data in the Protein Identification Resource (PIR). This group has recently been joined by others in the Federal Republic of Germany and in Japan.

The archive of three-dimensional structures of biological macromolecules is the Protein Data Bank at Brookhaven National Laboratory in New York, U.S.A. It collects the results of structure determinations, primarily by x-ray crystal structure analysis, but with a soupçon of structures determined by neutron diffraction; these to be joined by structures determined by NMR, which has established itself as quite a fruitful source of structural information for relatively small macromolecules. The Crystallographic Data Center in Cambridge, England, maintains a database of small molecular structures determined by x-ray crystallography. This information is extremely useful in studies of the conformations of the component units of biological macromolecules, and for investigations of macromolecule-ligand interactions.

A Task Group of CODATA (Committee on Data of the International Council of Scientific Unions), chaired by Prof. B. Keil of the Institute Pasteur; secretary, Dr. A. Tsugita of the Science University of Tokyo, has been working to try to foster collaboration among data banks, and between the data banks and the scientific community.

## Data Distribution

In the past, most of the data banks distributed their contents on magnetic tape or floppy disk, emitting successive releases at standard intervals, typically 3 months apart. Recently there has been some exploration of the use of computer networks for data distribution (as well as for submission of data), using the concept of a Netserver which responds to queries sent in over networks by returning a requested item as a reply (if possible). Other high-density storage media—notably CD-ROM—are also being explored; particularly exciting is the possibility of desk-top information retrieval systems self-contained in a personal computer-CD reader combination.

## Information-Retrieval Systems

To say that an understanding of the relationships among the data will provide the keys to breakthroughs in theoretical and experimental biology raises more questions than it answers. The availability of so much data, and the large number and incomplete definition of the relationships among them, create serious problems of data storage, quality control, and information retrieval. Plans for “mega-projects” such as the sequencing of the entire human and rice genomes will only intensify these challenges.

The rapid increase *of computer* power in *recent* years has begun to give us the tools to address these problems, and we must focus on the problems of design of a system to store, check, update, and distribute the incoming data, and then to provide the tools to produce new scientific results. These problems are common to many fields of science. Their general solution, based on an effective data base management system, has advantages that are well known: Applications programmers are relieved from standard management tasks, quality control is easier— redundancy and inconsistency in the data or in its formatting can be reduced, and the integrity of the data thereby more easily maintained.

A molecular biology information system must include both sequence and structural data, and the database management system must be able to answer, directly, most of the questions that investigators want to ask about the relationships among the data. It must also be flexible enough to accommodate new questions. Whether a commercial database management system can be used, or whether modifications or extensive redesign are required, depends on the structure and type of the information, on the manipulations to be performed on the data, and on the universe of user queries. Because the field is evolving with great speed intellectually, it is very hard to foresee the kind of questions we will come up with, even in the next few years. In the design of a database management system for molecular biology it would be fatal to sacrifice flexibility for efficiency.

It is necessary to present structural and non-structural data in the same framework. Inquiries such as “Is this sequence fragment present in other sequences?” and “Is this structural motif present in other structures?” should be asked by the user within a common framework of dialog. Of course the second type of question is more complicated, for it requires an interface general enough to define a structural motif. Thus the question “Is this new entry similar to any already-existing one?” is relatively well-defined if we are talking about linear (sequence) data, because the answer can be expressed in terms of the number of common residues and standard statistical parameters. In contrast, the same question, in the sense of structural similarity, requires further specification: For example, do we want to know about the overall shape of the molecule, about the relative positions of secondary structure elements, about the geometry of conserved residues in the active site? All these are legitimate inquiries, and such variations place great strain on a query interpreter.

Proteins exhibit both complex topological features and detailed local structural patterns. The careful observation of proteins, one at a time, can help us to define and propose some general principle of protein architecture; the comparative analysis of several structures probe the likelihood that our hypothesis is correct, and devising a general algorithm for testing the hypothesis with the entire available data set can confirm the proposed rule.

A problem facing those who would design a “packaged” system is the difficulty of defining a set of operations that encompasses the needs of the users. We ourselves, after having spent years in analyzing the operations useful in research (trying with only limited success to define a set of “elementary” operations in terms of which most manipulations might be defined) and in constructing software, find it frustrating that when a new project is undertaken it almost always requires the development of new tools.

Thus the database will have to cope with all the problems well-known from traditional “one-dimensional” databases, and also new ones specifically related to a “three-dimensional” database and a subject with widening intellectual horizons. The traditional problems include, for example, the problems of accommodating uncertain and partial data, of updating the system without loss of continuity, and most important of all, the problem of checking the data for consistency. Particular to chemical structural data is the need for a molecular graphics interface. Because features of the database entries must be presented graphically, in a consistent way, the design of the database must include a means of integrating the retrieval of information with molecular graphics packages.

A complete database should provide a flexible graphics interface allowing the user to visualize the atomic details of protein and nucleic acid structures, and some schematic view of their overall shape and secondary and tertiary structure. Whether the interface should include the graphics software or provide a way of interchanging information with the several existing graphics packages is not the most important question. The objection that in the first case the database would be more hardware-dependent will be overcome by the spread of graphics standards. What is needed in both cases is the availability of a clear definition of objects, representations and views applied to molecular objects.

## Molecular Graphics

How do we go about analyzing protein structures? First, we make a general inspection of the structures, using computer graphics. Programs take a set of coordinates and create a visual image on some device. The system gives the user the facility of selecting a portion of the molecule to be shown, selecting the orientation of the picture, and selecting the representation of the structure.

Two basic representations are (1) to show each atom as a sphere, distinguishing different atoms by different colours or shades; (2) to show each bond as a line. The former requires what is called a “raster” device, giving an image with the appearance of a television screen. Typically the image can contain 512×512 or 1024×1024 “pixels,” with each point chosen from one of 256 (or more) possible combinations of colour and intensity; thus one might have 16 intensity levels of each of 16 colours.

The second type of representation is called line or vector or calligraphic graphics. Here the technology exists to draw tens of thousands of lines, in different colours, at a refresh speed that does permit real-time rotation, which greatly enhances the observer’s perception of the three-dimensional structural relationships. (Real-time rotation of raster pictures is possible in the new generation of graphics workstations, which are now being applied to molecular biology. This capacity has existed for some time, but until recently only in devices of such high cost that they were limited to special applications such as the training of aircraft pilots.) Other important methods of enhancing the perception of three-dimensional structural relationships include stereo, hidden-line removal, and depth cueing (that is, the diminishing of intensity of objects farther from the eyepoint.)

**Figure 1:**
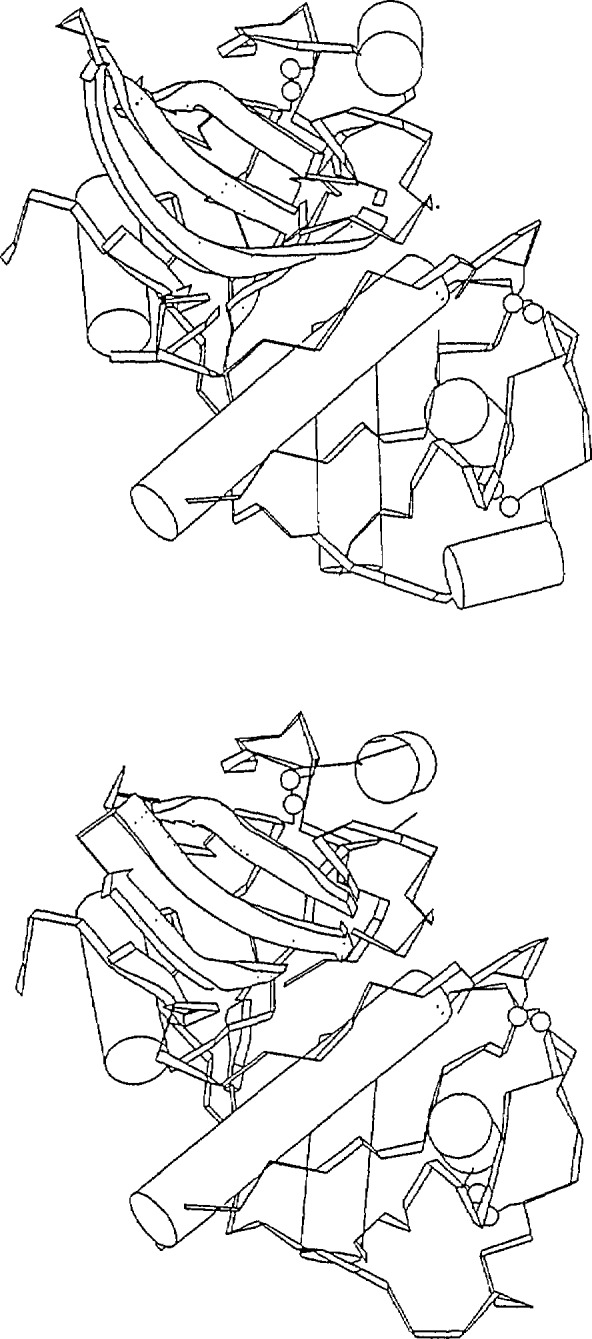
Two closely-related proteins; (a) actinidin [7] and (b) papain [8]. The amino acid sequences of these molecules have about 50% identical residues.

**Figure 2:**
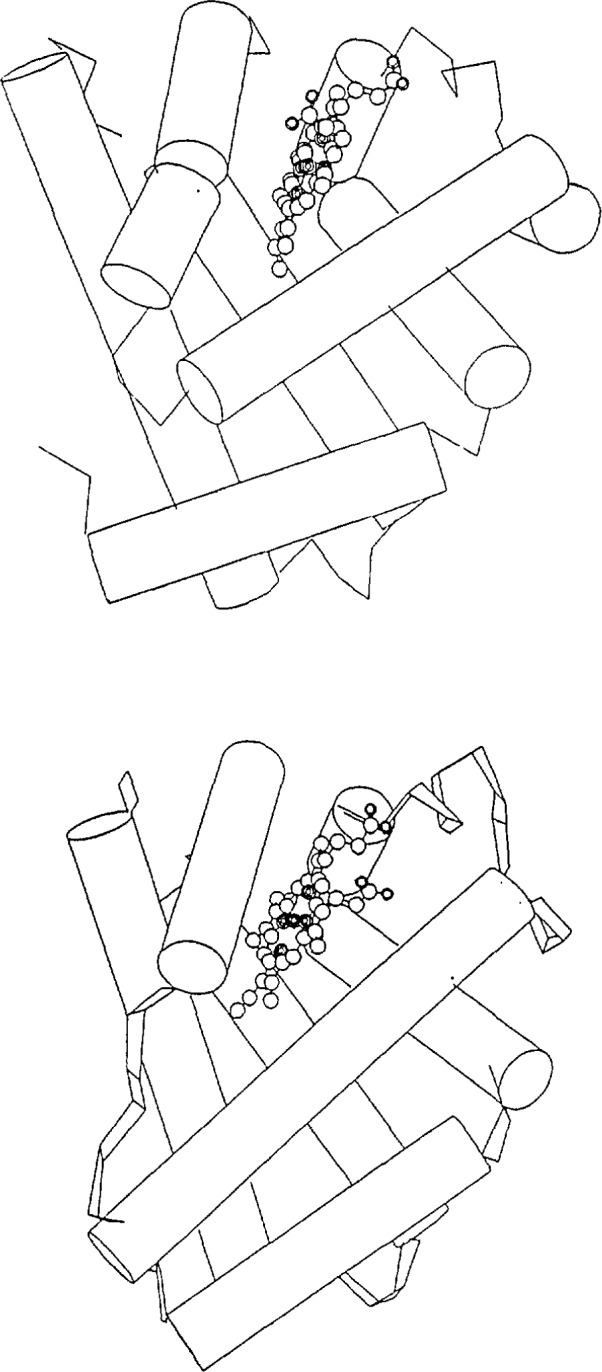
Two quite distantly-related proteins: (a) sperm whale myoglobin [9] and (b) lupin leghaemoglobin [10]. In this case almost the entire chains have the same fold.

**Figure 3:**
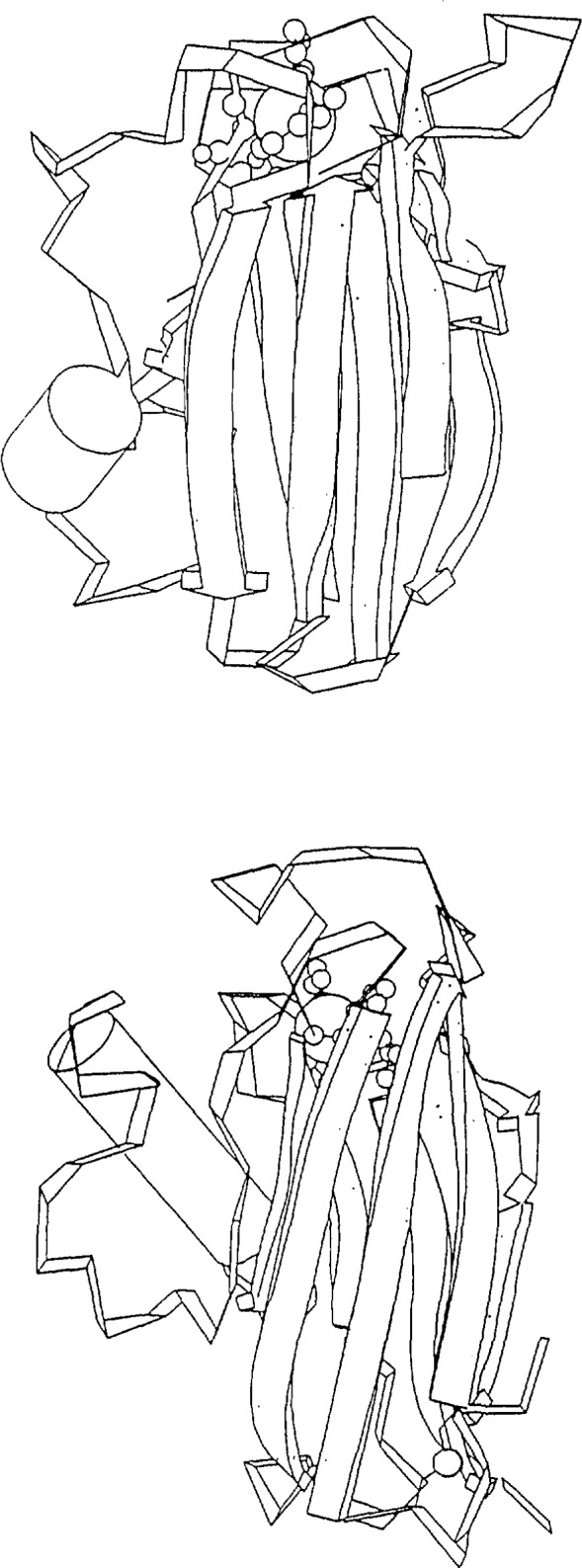
Two other distantly-related proteins: (a) poplar leaf plastocyanin [11] and (b) *A. denitrificans* azurin [12]. In this case the double *β*-sheet portion of these molecules retains the same fold, but the long loop at the left changes its conformation completely.

Because of the complexity of protein structures, pictures in which every atom or every bond is shown individually are often uninformative. People have therefore devised simplified or schematic representations. In these, a common grouping of atoms called an *α*-helix may be shown as a cylinder, and another common grouping of atoms called a strand of *β*-sheet may be shown as a large arrow (see figs. 1–3).

The analysis of a protein into helices, sheets, and other regions (often called loops) is part of the initial investigation of the structure and might be considered analogous to the parsing of a sentence, or at least to the identification of nouns and verbs. Helices and sheets are common arrangements of regions of proteins, stabilized by hydrogen bonds. They were predicted by Linus Pauling on the basis of physico-chemical principles before the discovery of the first protein structures, myoglobin and haemoglobin, in which the presence of helices was gratifyingly confirmed.

We can identify helices and sheets in proteins either visually, or by the detection of hydrogen bonds by purely numerical analysis of the coordinates, or by geometrical analysis of the positions of the atoms. There exist programs that will take a set of coordinates and produce a set of helix and sheet assignments automatically. Because of the not uncommon “fraying” of the ends of these regular substructures, these programs work fairly well but not perfectly.

Knowing where in the structure the helices and sheets lie, we can create a variety of representations of the structure. Protein structures have been classified into certain basic types on the basis of the types of secondary structures they contain and the spatial relationships between them. Such a diagram will be enough for an expert to place a new structure in the current scheme, or to recognize a real novelty.

Storing images or the coordinates necessary to rebuild them could be transparent to the user if a “molecular graphics metafile” is provided, where a clear definition of the properties of the displayed object is stored. While standardizing the graphic representation of a molecular object is relatively straightforward when dealing with one molecule at a time, several problems arise when more than one molecule has to be displayed in the same coordinate space. Showing two superimposed molecules, or an enzyme together with its substrate, requires in both cases the visualization of two molecules, but the physical meaning of the two double images is completely different. Some operations are allowed in one case (for example in the first case two atoms occupy the same position in space) but forbidden in the other. In other words the “metafile” should also define the possible operations that can be performed in each case, so that the application program, whether it is a part of the database or not, should treat the two cases differently providing the user with the appropriate functions for each.

## Protein Modeling

The general ideas presented here can now be illustrated with an important example; the question of modeling the structures of unknown proteins. In order to have a specific framework for this discussion, let us consider a particular problem; one which in fact arises in virtually this exact form.

Suppose we know the structures of two related proteins, for example, the sulphydryl proteases actinidin [7] and papain [8], or the two electron-transport proteins plastocyanin [9] and azurin [10], or sperm whale myoglobin [11] and lupin leghaeraoglobin [12] (see figs. 1–3). Suppose someone shows up with a third sequence, of a natural protein of unknown structure related to the other two. What can we say about its structure? (The restriction to natural variants is now important because molecules synthesized in the laboratory have not undergone the trial of natural selection and may not follow the same rules.)

In order to answer this question, we must know how to align the sequences of the known proteins, we must be able to identify and describe the structural differences between the known proteins, and we must be able to know how the differences in the amino acid sequences are related to the structural differences. Deriving this insight from the known proteins we can extrapolate to their unknown relative. Let us consider some of the computational steps we go through, and the nature of the software and hardware that have proved useful.

Let us first dispose of what we might with some temerity call a potential distraction: Someday it may be possible to ignore the fact that the unknown protein is related to others of known structure, and to predict its conformation from physical principles. This is just not possible today (see below).

It follows that, given a new sequence, we must first try to find out whether it is related to proteins of known structure. There are now fairly standard techniques for screening databases of sequences, to pick up many—but not all—relationships. Very distant relationships may elude these procedures, as it is a fact that structural relationships can exist when the overall sequence similarity has diverged so far as to conceal the homology. There are more sensitive methods for picking up members of some individual protein families, by looking for a specific “fingerprint” or “signature” of a protein that may involve only a small fraction of the residues.

If we find that the unknown protein is related to other proteins of known structure, it is possible to draw two conclusions about its conformation:

1. the structure of the unknown protein is like the structure of the known proteins and
2. the structure of the unknown protein is unlike the structure of the known proteins.

Although this sounds like something from Alice in Wonderland, both comments are true. The first is a statement that related amino acid sequences determine protein structures that have the same general topology or fold, over at least 50% of the molecule. The second statement points out that amino acid sequence changes produce conformational changes, so that the structure of one of the proteins will be a distorted version of the structure of the other. The extent of the distortion, which limits the quality of the model of the unknown protein that we could build, depends on how far the amino acid sequences have diverged.

We have already discussed how we look at one protein structure at a time. To reason about an unknown protein from related known ones, we must now turn to the question of how we analyze the structural differences between two related proteins.

There is a basic computational tool in comparative structural analysis, which is the geometric superposition of a pair of structures. Given two lists of atoms, which may be regions selected from two proteins, the problem is to find a rotation matrix and translation vector that will optimally superpose the two structures, in a least-squares sense. We must know the proper correspondence of the atoms in the two structures, not a trivial question in the face of insertions and deletions of amino acids in the sequences of proteins.

Fortunately, this is a very simple problem to solve, and several fast and reliable algorithms are available. The result of such a calculation is the optimal geometric transformation, and the root-mean-square (rms) deviation of the atomic positions. It is also possible to list individual atomic deviations and thereby distinguish well-fitting regions from other regions in which structural change has occurred. The operation of performing superpositions of selected regions of proteins is the basic tool of quantitative structural comparison, akin to something as fundamental as pipetting in the laboratory.

What does such analysis tell us about the structural differences between pairs of related proteins? First, it shows that in a family of proteins there is a core of the structure that retains the same basic topology, or fold, and the rest can have a completely different conformation [1,2]. (To explain the idea of the common core of two structures, look at the letters B and R. Considered as structures they have a common core corresponding to the letter P. Outside the common core they differ: at the bottom right B has a loop and R has a diagonal stroke.) In plastocyanin and azurin, the double *β*-sheet retains its fold but the long loop at a side of the sheet does not. Secondly, it shows that although individual helices and sheets tend to retain their structures fairly rigidly, there are changes in their relative geometrical relationship—shifts and rotations of one relative to another. Using superposition calculations we can measure the magnitude of these shifts and rotations.

What can we then say about the structure of a new protein? The general comment is that the common core that this protein shares with the known structures will have the same fold; but, except in special cases, we cannot predict the structure of the regions outside the core. More specifically, we can relate the fraction of the structure in the core, and the magnitude of the distortions of the core structure, to the divergence of the amino acid sequences. Note that these quantitative results required numerical superposition calculations, not merely looking at the structures. There are numerous program systems that combine interactive graphics with superposition facilities.

The basic rule-of-thumb that emerges from these results is that if the amino acid sequences are 50% identical, or more closely related, it will be possible to build a useful model, by transferring the side chains of the new sequence to the backbone of the most closely-related protein of known structure, retaining the side chain conformation whenever possible. In these circumstances, the model will be expected to have the correct fold in over 90% of the structure, and the overall rms deviation of the backbone will be no more than 1 Å. If the sequences are more closely related, the model will be correspondingly better. Such a model would be of a quality useful for interpreting changes in function.

If the amino acid sequences of the new protein and that of its closest relative of known structure have lower than 50% residue identify, we should be more discouraging about building a useful model. If the sequences have only 20% residue identity, the model might even have the correct fold in only half of the structure, and the atomic deviations of the remaining core might well be over 2 Å. Most people would feel that such a model would not be a useful guide to interpreting the properties of the unknown protein. However, often the binding site of a protein family is better preserved than the rest of the protein structure, and it may be possible to interpret changes in specificity in terms of mutations in and around the binding site itself.

It will have been noticed that our model building procedure has been the most naive and conservative possible: we identify the closest relative of the known structure, and retain as many structural features of this known structure as possible. Many people have suggested that this should be regarded as only a zero-order model, and that more powerful computational techniques might improve it. Such techniques might produce a quantitative improvement in the prediction of the conformation of the common core, or yield useful predictions of portions of the structure outside the core.

To achieve a global improvement in the structure, many people have tried to apply energy minimization or molecular dynamics. These are general methods, based on a detailed quantitative representation of the physical forces involved, to predict the conformations that these forces would create. It has been known for some time that these methods cannot fold up a protein “from scratch”. It has more recently become clear that these methods cannot substantially improve a model of the type constructed as we have described. The problem seems to be, that if you take a native protein structure, just as determined by x-ray crystallography, and subject it to energy minimization, the program will find that the experimental structure is not at an energy minimum, and the minimum-energy conformation found will have an rms deviation from the correct structure of about 1 Å. But this is as large or larger than the deviation of the naive model, so significant progress has not been made.

Energy minimization is useful for “tidying up” a structure, for example, closing up gaps in the chain resulting from deletions in the amino acid sequence. But it does not give an effective way to move a model towards the correct structure.

The, question of modeling portions of the structure outside the core is one in which some progress has been made, at least for relatively short loops. We have faced the problem of modeling the antigen-binding loops of antibodies [3,4]. Here and in related cases, the effective approach has been to look in the general corpus of known structures for a prefabricated piece that will fit. For hairpin loops between consecutive strands of a *β*-sheet, there are certain rules relating the conformation of the loops to the length and sequence of the loop [4,5]. In favourable cases, these rules guide us in building the conformation of a loop by stitching in a piece from a known structure.

A more general approach, developed by T. Alwyn Jones and colleagues, is based on a general method of substructure search. This may be thought of as roughly analogous to a standard editing operation: that of identifying occurrences of a character string in text. The basic idea is that if one has fixed two points in the chain, perhaps as the ends of regions of secondary structure in the core, one can extract from a database of known structure examples of regions that match the structure of the two ends, and then can look at what appears in between. In favourable cases, it will emerge that there is a preferred way to connect the two regions and this can be applied to the modeling of the loops.

## Conclusions

Advances in experimental techniques have presented us with much knowledge from our biological heritage, in a form accessible to computer data banks and information retrieval systems. The problem we currently face is to provide the interface between the archived data and the practicing scientist, so that this knowledge can be fruitful and multiply.
